# Prognostic Significance of Neutrophil Lymphocyte Ratio in Patients with Gastric Cancer: A Meta-Analysis

**DOI:** 10.1371/journal.pone.0111906

**Published:** 2014-11-17

**Authors:** Xi Zhang, Wei Zhang, Li-jin Feng

**Affiliations:** 1 Department of Pathology, Shanghai Tenth People's Hospital, Tongji University, School of Medicine, Shanghai, China; 2 Department of Medical Oncology, Shanghai Tenth People's Hospital, Tongji University, School of Medicine, Shanghai, China; National Cancer Center, Japan

## Abstract

**Background:**

Several studies have shown that neutrophil lymphocyte ratio (NLR) may be associated with the prognosis of gastric cancer (GC), but the results are controversial.

**Methods:**

This study was performed to evaluate the prognostic implications of neutrophil lymphocyte ratio of GC in all available studies. We surveyed 2 medical databases, PubMed and EMBASE, to identifyall relevant studies. Data were collected from studies comparing overall survival (OS), disease-free survival (DFS) and progression-free survival (PFS) in patients with GC.

**Results:**

Ten studies (n = 2,952) evaluated the role of NLR as a predictor of outcome were involved for this meta-analysis (10 for OS, 3 for DFS, and 2 for PFS). Overall and disease-free survival were significantly better in patients with low NLR value and the pooled HRs was significant at 1.83 ([95% CI], 1.62–2.07) and 1.58 ([95% CI], 1.12–2.21), respectively. For progression-free survival, the pooled hazard ratio of NLR was significant at 1.54 ([95% CI], 1.22–1.95). No evidence of significant heterogeneity or publication bias for OS and DFS was seen in any of the included studies.

**Conclusion:**

This meta-analysis indicated that elevated NLR may be associated with a worse prognosis for patients with GC.

## Introduction

Despite the incidence of gastric cancer is decreasing, it remains one of the most frequent causes of cancer-related death worldwide [1]. The incidence of gastric cancer varies widely in different regions and is particularly common in East Asia [2]. In china, where gastric cancer is endemic, more patients are diagnosed in middle or late stage, which is reflected by poor overall survival rates. Although there have been great improvements in diagnostic and treatment technologies, most of the gastric patients still have either regional or distant metastatic disease with the 5-year overall survival less than 10% [3]. Therefore, it is important to identify prognostic factors for these patients in order to select patients for tailor treatment. Up to now, the prognosis significance of lymph node status [4], depth of tumor invasion [5] and macroscopic tumor size [6] are well known in GC. In addition, elevations of serum tumor markers can also be an independent predictor of adverse prognosis [7]. However, none of these have been demonstrated to be sufficiently effective for clinical use. More recently, established systemic inflammation-based prognostic scores have been explored extensively, such as NLR and serum C-reactive protein (CRP). CRP is an acute-phase response protein, which has been proven to be an independent prognostic factor for survival in malignancy [8]. However, CRP is not routinely measured in many hospitals, and CRP level displays nonspecific change after treatment [9]. NLR can be suggested as the balance between pro-tumor inflammatory status and anti-tumor immune status. Patients with elevated NLR have a relative lymphocytopenia and neutrophil leukocytosis in favor of protumor inflammatory response, which gained its prognostic value in patients with colorectal cancer [10], lung cancer [11], pancreatic ductal adenoma [12], etc. Elevated level of NLR in GC patients may predict poorer clinical outcome [13], while some authors did not agree with the former results [14]. The aim of this study was to comprehensively and quantitatively summarize the global results to evaluate its prognostic value for patients with GC.

## Methods

### Search strategy and eligibility criteria

This meta-analysis was executed in accordance with the Preferred Reporting Items for Systematic Reviews and Meta-Analyses (PRISMA) guidelines [15]. A systematic literature search of relevant studies was conducted in PubMed and EMBASE up to June 2014. We used the following search terms without restrictions: “NLR”, “neutrophil to lymphocyte ratio”, “neutrophil lymphocyte ratio”, “prognosis” and “gastric cancer” or “GC”. Moreover, reference lists of retrieved articles were also reviewed to identify any studies that were not identified from the preliminary literature searches. Studies were included if they met the following criteria: (1) patients with gastric cancer in the studies were histopathologically confirmed (2) neutrophil-lymphocyte ratio values were reported (3) they evaluated the corelation between neutrophil lymphocyte ratio and the survival outcome of GC and (4) if studies' hazard ratios (HRs) were not directly repored, estimation of the HR could be reconstruct by other data. Articles were excluded from the meta-analysis based on the following criteria: (1) letters, conference abstracts, editorials, review articles, not full text in English, studies on cancer cell and animal model and irrelevant studies (2) studies had overlapping or duplicate data (3) studies failed to present the cut-off value for elevated NLR.

### Quality Assessment

The quality of studies was assessed according to Newcastl-Ottawa Quality Assessment Scale (NOS) [16] by two reviewers (Xi Z and Wei Z). This scale includes three aspects of evaluation: selection, comparability, and outcome between the case group and control group. Studies that scored ≥6 were assigned as high-quality studies. Any disagreement was resolved by discussion.

### Data extraction

Two investigators independently evaluated and extracted the data. All studies were double-checked by both and disagreements were resolved by consensus. The extracted data elements of this review included the following: (1) publication details, including first author's last name, publication year, and origin of the studied population (2) characteristics of the studied population, including sample size, age, and stage of disease and (3) HR of NLR for OS, DFS and PFS as well as their 95% CIs and p values and (4) follow-up time (5) cut-off values for elevated HR. If data for HR was not available, we extracted the total numbers of observed deaths and the numbers of patients in each group to calculate HR. Data were extracted from the graphical survival plots when data were only available as Kaplan-Meier curves [17]. If several estimates were reported in the same article, we chose the most powerful one (multivariate analysis was superior to univariate analysis).

### Statistical Analysis

HRs and their 95% CIs from each study were used to calculate pooled HRs. The heterogeneity of the combined HRs was performed using Cochran's Q test and Higgins' I-squared statistics. A P value <0.05 was considered significant. We used the random effects model (Der Simonian and Laird method) if heterogeneity was observed (P<0.05). The fixed effects model was applied in the absence of between-study heterogeneity (P≥0.05) [18]. Publication bias of literature was evaluated using Begg's funnel plot and the Egger's linear regression test and a p<0.05 was considered significant. Statistical analyses were carried out using the statistical software Stata (version 12.0).

## Results

### Literature search

**Figure 1:**
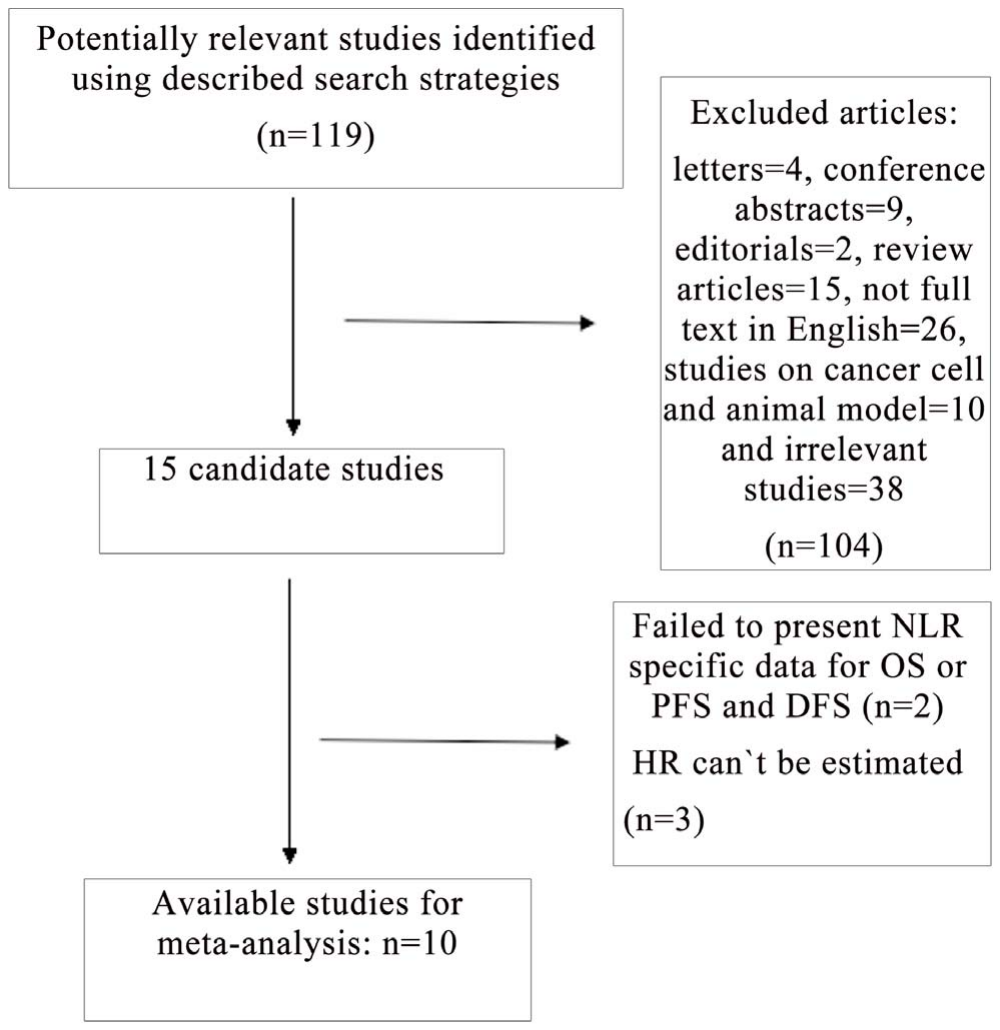
Flow chart of the meta-analysis.

A flow diagram of our literature search is shown in Figure 1. We identified 15 potentially relevant articles concerning NLR and prognosis of gastric cancer. Three studies were excluded as HR can't be calculated by the described method [19]–[21] and 2 studies were excluded as failed to present NLR specific data for OS or DFS and PFS [22]–[23]. A total of 10 articles [13]–[14], [24]–[31] that met the inclusion and exclusion criteria were retrieved. Of these reports selected for further evaluation, 10 investigated the prognostic role of NLR for OS, 3 for DFS and 2 for PFS, respectively.

### Study Characteristics

The characteristics of the included studies were summarized in Table 1. We collected the data from 10 studies, which involved a total of 2,952 patients from the Korea, China, Japan, Italy and Turkey. Treatment methods for nine studies were surgery and chemotherapy. Patients of one study were treated by multiple-therapy. Four studies enrolled less than 200 patients and six studies had more than 200 patients. The risk of bias in included studies was outlined based on the Newcastle-Ottawa Quality Assessment Scales. (Table 2) HR and 95%CI were reported directly in 9 of the enrolled cohorts. Jung et al presented separate cut-off value with cut-off value = 3.0 for OS and cut-off value = 2.0 for DFS. In the study by Jin, HR and its 95% CIs before treatment was calculated from Kaplan-Meier curves.

**Table 1:** Main characteristics of all the studies included in the meta-analysis.

First author, date(ref.)	Study region	No (M/F, n)	Treatment(predominant)	Follow-up(M) (median and range)	Age(ys) (median and range)	NO. of Distal metastasis	Survival Analysis	Cutoff Value(<CV/≥CV)	HR	Summary results	Clinical stage	(III+IV)/All (n%)	Outcome
Lee et al.(2013)	Korea	174(114/60)	chemotherapy	14.9(1–47.9)	18–79	104	prospective	3.0(112/62)	R	positive	I–IV	145/174(83.3%)	OS
Wang et al.(2012)	China	324(225/99)	surgery	39.9(23.8–57.4)	NR	0	retrospective	5.0(313/11)	R	negative	III	324/324(100%)	OS/DFS
Jeong et al.(2012)	Korea	104(69/35)	chemotherapy	11.9(10.2–11.9)	52.5(28–82)	104	retrospective	3.0(49/55)	R	positive	IV	104/104(100%)	OS
Jung et al.(2011)	Korea	293(193/100)	surgery	38.2(4.2–65.5)	63(21–96)	120	retrospective	2.0(138/155)	R	positive	III–IV	293/293(100%)	OS/DFS
Shimada et al.(2010)	Japan	1028(709/319)	surgery	23(12–84)	65(26–89)	27	retrospective	4.0(127/901)	R	positive	I–IV	312/1028(30.3%)	OS
Jin et al.(2013)	China	46(36/10)	Chemotherapy	NR	60(37–77)	6	prospective	2.5(26/20)	E	negative	I–IV	34/46(73.9%)	OS/PFS
Aurello et al.(2014)	Italy	102(62/40)	surgery	40.8(8–107)	69±10.6	0	retrospective	5.0(74/28)	R	negative	I–IV	53/102(51.9%)	OS/DFS
Cho et al.(2014)	Japan	268(175/93)	chemotherapy	11.3(2.4–57.8)	55.4±12.48	187	retrospective	3.0(130/138)	R	positive	IV	268/268(100%)	OS/PFS
Jiang et al.(2014)	China	377(253/124)	surgery	42(1–103)	64±11.7	0	prospective	1.44(68/309)	R	positive	I–III	236/377(62.5%)	OS
Dirican et al.(2013)	Turkey	236(162/74)	multiple therapy	NR	58(30–86)	105	retrospective	3.8(147/89)	R	positive	I-IV	210/236(88.9%)	OS

OS: overall survival; DFS: disease-free survival; PFS: progression-free survival; NR: not reported; R: reported; E: estimated; HR: hazard ratio; CV; cutoff value; Assessment Scale; All: all patients.

**Table 2:** Quality Assessment of included studies based on the Newcastle-Ottawa Scales.

Study	How representative was the exposed Cohort	Selection of non-exposed cohort	Ascertainment of exposure	Demonstration that outcome of interest was not present at start of study	Of cohorts on basis of design or analysis	Assessment of outcome	Follow up Long enough for outcomes to occur	Adequacy of cohort follow-up
Lee et al.(2013)	Somewhat representative of GC patients	Drawn from the same community as exposed cohort	Written self-report	Yes	Study controls for multiple covariate	confirmation of the outcome by reference to secure records	Yes	No description
Wang et al.(2012)	Somewhat representative of GC patients	Drawn from the same community as exposed cohort	From structured interview	Yes	Study controls for multiple covariate	confirmation of the outcome by reference to secure records	Yes	No description
Jeong et al.(2012)	Somewhat representative of GC patients	Drawn from the same community as exposed cohort	Secure record	Yes	Study controls for multiple covariate	confirmation of the outcome by reference to secure records	Yes	Complete follow-up
Jung et al.(2011)	Somewhat representative of GC patients	Drawn from the same community as exposed cohort	Secure record	Yes	Study controls for multiple covariate	confirmation of the outcome by reference to secure records	Yes	No description
Shimada et al.(2010)	Representative of GC patients	Drawn from the same community as exposed cohort	From structured interview	Yes	Study controls for multiple covariate	confirmation of the outcome by reference to secure records	Yes	No description
Jin et al.(2013)	Somewhat representative of GC patients	Drawn from the same community as exposed cohort	Secure record	Yes	Study controls for multiple covariate	confirmation of the outcome by reference to secure records	No	No description
Aurello et al.(2014)	Representative of GC patients	Drawn from the same community as exposed cohort	From structured interview	Yes	Study controls for multiple covariate	confirmation of the outcome by reference to secure records	Yes	Complete follow-up
Cho et al.(2014)	Somewhat representative of GC patients	Drawn from the same community as exposed cohort	Secure record	Yes	Study controls for multiple covariate	confirmation of the outcome by reference to secure records	Yes	No description
Jiang et al.(2014)	Somewhat representative of GC patients	Drawn from the same community as exposed cohort	Secure record	Yes	Study controls for multiple covariate	confirmation of the outcome by reference to secure records	Yes	No description
Dirican et al.(2013)	Somewhat representative of GC patients	Drawn from the same community as exposed cohort	Secure record	Yes	Study controls for multiple covariate	confirmation of the outcome by reference to secure records	No	No description

### Outcome from eligible studies

**Figure 2:**
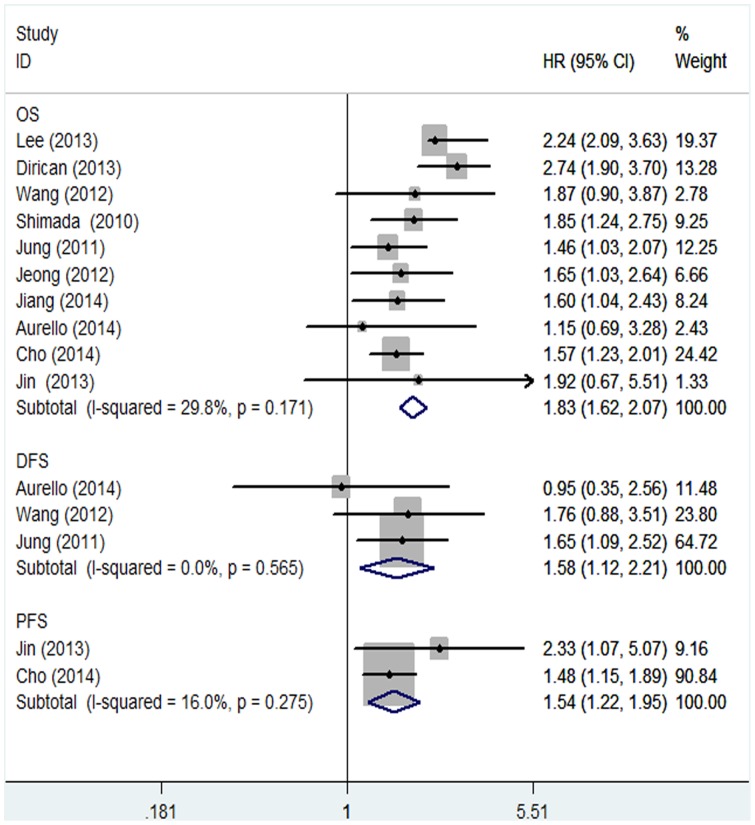
Forest plots of studies evaluating hazard ratios (HR) with 95% confidence interval (95% CI) for high NLR levels as compared with low levels. Survival data are reported as overall survival, disease-free survival and progression-free survival.

In the 10 studies evaluating OS, there was no significant heterogeneity between studies for categorized NLR (I-squared = 29.8%; p = 0.171). The fixed-effect model was applied to calculate the pooled HR, and its 95% CI. The pooled HR of 1.83 (95%CI: 1.62–2.07) indicated that patients with elevated NLR have shorter OS (Figure 2).

Subgroup analyses was conducted for OS. Subgroup analyses by treatment methods showed that elevated NLR predicted poor prognosis in patients treated with both surgery and chemotherapy [(HR = 1.59, 95%CI: (1.30–1.95); HR = 1.82, 95%CI:(1.53–2.15)]. Stratification by sample size, we found the pooled HRs was 1.77, 95%CI: (1.54–2.05) for studies with more than 200 cases and 1.97, 95%CI: (1.58–2.46) for studies with less than 200 cases. The results revealed that high NLR remained to be a worse prognostic marker regardless of sample size. Stratification by cut-off value≤3.0 and cut-off value>3.0, it was found that the pooled HRs was still a poor predictor for GC [HR = 1.72, 95%CI: (1.49–1.99)] for cut-off value≤3.0 and [HR = 2.14, 95%CI: (1.70–2.70)] for cut-off value>3.0. In the subgroup analyses by geographic region, we found that elevated NLR was still a poor predictor for eastern patients [HR = 1.74, 95%CI: (1.53–1.99)] but not for western patients [HR = 1.80, 95%CI: (0.65–4.97)]. When performing subgroup analyses stratified by TNM stage, we found that increased NLR was a negative predictor in patients with stage III or IV[HR = 1.57,95%CI: (1.31–1.87)]and patients with stage I–IV[HR = 2.09,95%CI: (1.77–2.47)], however, NLR might be a more important prognostic factor for early TNM stage patients with the higher pooled HRs (Table 3).

**Table 3:** Summary of the subgroup meta analysis results for OS.

Subgroup	N	Random-effects model HR(95%)CI	Fixed-effects model HR(95%)CI	Heterogeneity	
				I^2^(%)	P Value
Treatment method					
Surgery	5	1.59(1.30–1.95)	1.59(1.30–1.95)	0	0.808
Chemotherapy	4	1.82(1.48–2.23)	1.82(1.53–2.15)	21	0.284
Sample size					
Sample size <200	4	1.94(1.52–2.47)	1.97(1.58–2.46)	7.3	0.357
Sample size≧200	6	1.79(1.46–2.20)	1.77(1.54–2.05)	44.4	0.11
Cut-off value					
Cut-off value>3	4	2.01(1.44–2.80)	2.14(1.70–2.70)	42.7	0.157
Cut-off value≦3	6	1.72(1.49–1.99)	1.72(1.49–1.99)	2.9	0.398
Geographic region					
Eastern countries	8	1.74(1.53–1.99)	1.74(1.53–1.99)	0	0.625
Western countries	2	1.80(0.65–4.97)	2.46(1.80–3.38)	74.5	0.048
TNM stage					
(III+IV)/All = 100%	4	1.57(1.31–1.87)	1.57(1.31–1.87)	0	0.936
(III+IV)/All<100%	6	2.03(1.65–2.21)	2.09(1.77–2.47)	28.7	0.220

HR: hazard ratio; CI: confidence interval; All: all patients.

Meta-regression was also conducted to explore the potential source of heterogeneity. The results showed that TNM stage (p = 0.049) may contribute to the source of inter-study heterogeneity.

For DFS and PFS, there were three studies reporting the data of NLR for DFS and two studies reporting the data of NLR for PFS in GC patients. The *P* values between-study heterogeneity for DFS and PFS were (I-squared = 0%; p = 0.565) and (I-squared = 16%; p = 0.275), respectively. As for the ability to evaluate DFS, the combined HR of 1.58 (95%CI: 1.12–2.21) showed that the high NLR had significant relationship with DFS in GC (Figure 2). As illustrated in Figure 2, elevated NLR predicted poor prognosis for PFS whose pooled HR was significant at 1.54 (95%CI: 1.22–1.95).

### Publication Bias

**Figure 3:**
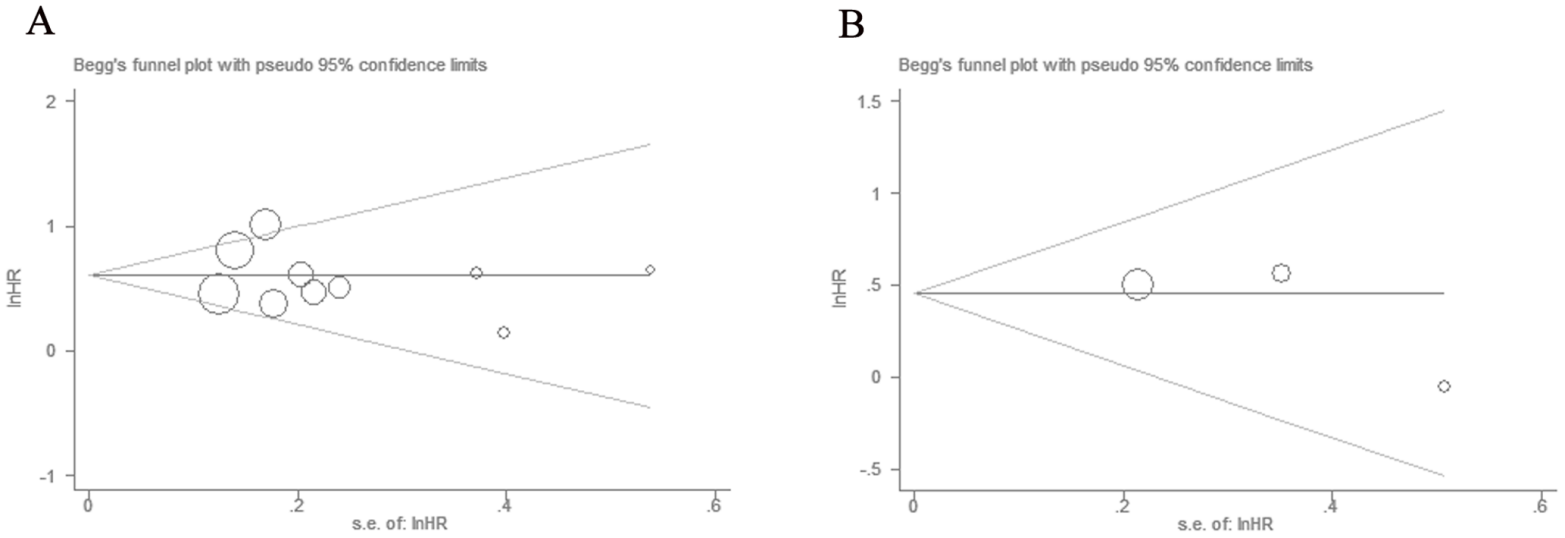
Funnel plots of studies included in the meta-analyses: A) overall survival, B) disease-free survival.

We applied funnel plots and Egger's test to evaluate publication bias of the included studies. As shown in Figure 3, the funnel plot was symmetrical. There was no evidence for significant publication bias for OS and DFS, since their p values for Egger were more than 0.1.

## Discussion

Our results from this meta-analysis including 10 studies with 2952 cases showed that elevated NLR was associated with OS, DFS and PFS. Subgroup analyses revealed that poor OS with high NLR could be found in patients treated with both surgery and chemotherapy. Elevated NLR was a significant prognostic marker to predict poor OS regardless of sample size and cut-off values. When subgroup was analyzed by geographic region, we found that elevated NLR was still a poor OS predictor for eastern patients but not for western patients. Moreover, when the prognostic significance of elevated NLR was evaluated by TNM stage, NLR might be a more crucial prognostic factor for early TNM stage patients.

Chronic inflammation is known to promote carcinogenesis contributing to the onset or progression of cancer [32]. Tumors can not only develop at the sites of inflammation, such as Helicobacter pylori infection is recognized as a causative agent for gastric cancer [33], but they can also trigger regional immune response and release inflammatory factors around the tumor which result in the formation of an inflammatory microenvironment. Inflammatory processes always accompany with progression of cancer, which can contribute to tumorigenesis by supplying cytokines, such as vascular endothelial growth factor (VEGF), interleukin-18 and matrix metalloproteinases [34]–[36] to the tumor microenvironment that promotes angiogenesis, and thus promotes tumor growth and metastasis.

In recent decades, a variety of predictors have been identified and applied for predicting GC outcomes. CEA, Her-2 are currently used in routine pathological assessment of GC. Ki-67, caspase-3 and p53 have also been reported associated with GC survival [37]. In addition, it is well known today that miRNAs have very important regulatory functions in cancer. Up to now, accumulating studies have investigated the diagnostic and prognostic values of miRNAs in GC. For example, Ueda T found that microRNAs are expressed differentially in gastric cancers and unique microRNAs are associated with progression and prognosis of GC [38]. However, the above-mentioned biomarkers should be examined in cancerous tissues. Thus it is impossible to monitor their levels continuously throughout disease progression. In contrast, NLR as an indicator of inflammation can be easily assayed in plasma or serum, which may be widely applied in the clinic.

NLR is known to possess prognostic value in cancer population. There are a number of possible mechanisms by which NLR is associated with worse outcome in patients with cancer. Firstly, the antitumor responses of natural killer cells and activated T cells may be suppressed by increased number of neutrophils around the tumor [39]. A high NLR reflects both a heightened neutrophil-dependent inflammatory response and a decreased lymphocyte mediated antitumor immune reaction, which may weaken the lymphocyte-mediated anti-tumor cellular immune response and contribute to aggressive tumor biology, cancer progression and poor prognosis. Secondly, circulating neutrophils contributes to tumor growth and progression by producing cytokines, such as tumor necrosis factor (TNF), IL-1, IL-6, and angiogenic factor vascular endothelial growth factor (VEGF) [40]. Thirdly, a reduced number of lymphocytes may weaken the lymphocyte-mediated anti-tumor cellular immune response. The neutrophil count alone may not reflect the prognostic information of a decreased lymphocyte mediated immune response, and a low lymphocyte count alone may not reflect the neutrophil driven tumor growth process. Hence, it is likely that the combined effects of neutrophilia and lymphocytopenia lead to a high NLR which may reflect the combined prognostic information of these two processes, and be a stronger predictor of outcome than either alone.

There are some limitations in this study. First, there is some heterogeneity of subjects for NLR in the OS group. Heterogeneity might be caused by characteristics of the patients, such as age, differentiation or disease stage, cut off values, treatment they might have received, the duration of follow-up, and adjustments for other cofactors. Moreover, our results are likely to be affected by the wide range of cutoff values for elevated NLR, which may affect the positive associations between NLR and GC prognosis. For example, cut-off scores of NLR were defined as 1.44, 2.5, 3.0, 4.0 or 5.0 by analyzing the ROC curve, median value or based on previous studies, however, subgroup analyses stratified by cut-off values showed that the NLRs prognostic value was not affected substantially. Second, the NLR is usually regarded as a prognostic marker in several diseases which are related to survival, such as cardiovascular diseases [41]. Thus, we cannot consider NLR as a “predictor” for survival unless the involved patients don't have other severe diseases related to NLR. Finally, only English studies were included in this analysis and small studies with null results tended not to be published, which may cause potential publication bias.

## Conclusions

In conclusion, our meta-analysis, including a quantified synthesis of all published studies, showed that elevated NLR was a poor predictor for survival in patients with gastric cancer. The critical role of NLR in cancer prognosis may contribute to its clinical utility. Considering the limitations of the present meta-analysis, further research with large-scale and standard investigations should be conducted.

## Supporting Information

Checklist S1 **PRISMA checklist.**
(DOC)
